# The novel Mechanical Ventilator Milano for the COVID-19 pandemic

**DOI:** 10.1063/5.0044445

**Published:** 2021-03-23

**Authors:** A. Abba, C. Accorsi, P. Agnes, E. Alessi, P. Amaudruz, A. Annovi, F. Ardellier Desages, S. Back, C. Badia, J. Bagger, V. Basile, G. Batignani, A. Bayo, B. Bell, M. Beschi, D. Biagini, G. Bianchi, S. Bicelli, D. Bishop, T. Boccali, A. Bombarda, S. Bonfanti, W. M. Bonivento, M. Bouchard, M. Breviario, S. Brice, R. Brown, J. M. Calvo-Mozota, L. Camozzi, M. Camozzi, A. Capra, M. Caravati, M. Carlini, A. Ceccanti, B. Celano, J. M. Cela Ruiz, C. Charette, G. Cogliati, M. Constable, C. Crippa, G. Croci, S. Cudmore, C. E. Dahl, A. Dal Molin, M. Daley, C. Di Guardo, G. D'Avenio, O. Davignon, M. Del Tutto, J. De Ruiter, A. Devoto, P. Diaz Gomez Maqueo, F. Di Francesco, M. Dossi, E. Druszkiewicz, C. Duma, E. Elliott, D. Farina, C. Fernandes, F. Ferroni, G. Finocchiaro, G. Fiorillo, R. Ford, G. Foti, R. D. Fournier, D. Franco, C. Fricbergs, F. Gabriele, C. Galbiati, P. Garcia Abia, A. Gargantini, L. Giacomelli, F. Giacomini, F. Giacomini, L. S. Giarratana, S. Gillespie, D. Giorgi, T. Girma, R. Gobui, D. Goeldi, F. Golf, P. Gorel, G. Gorini, E. Gramellini, G. Grosso, F. Guescini, E. Guetre, G. Hackman, T. Hadden, W. Hawkins, K. Hayashi, A. Heavey, G. Hersak, N. Hessey, G. Hockin, K. Hudson, A. Ianni, C. Ienzi, V. Ippolito, C. C. James, C. Jillings, C. Kendziora, S. Khan, E. Kim, M. King, S. King, A. Kittmer, I. Kochanek, J. Kowalkowski, R. Krücken, M. Kushoro, S. Kuula, M. Laclaustra, G. Leblond, L. Lee, A. Lennarz, M. Leyton, X. Li, P. Liimatainen, C. Lim, T. Lindner, T. Lomonaco, P. Lu, R. Lubna, G. A. Lukhanin, G. Luzón, M. MacDonald, G. Magni, R. Maharaj, S. Manni, C. Mapelli, P. Margetak, L. Martin, S. Martin, M. Martínez, N. Massacret, P. McClurg, A. B. McDonald, E. Meazzi, R. Migalla, T. Mohayai, L. M. Tosatti, G. Monzani, C. Moretti, B. Morrison, M. Mountaniol, A. Muraro, P. Napoli, F. Nati, C. R. Natzke, A. J. Noble, A. Norrick, K. Olchanski, A. Ortiz de Solorzano, F. Padula, M. Pallavicini, I. Palumbo, E. Panontin, N. Papini, L. Parmeggiano, S. Parmeggiano, K. Patel, A. Patel, M. Paterno, C. Pellegrino, P. Pelliccione, V. Pesudo, A. Pocar, A. Pope, S. Pordes, F. Prelz, O. Putignano, J. L. Raaf, C. Ratti, M. Razeti, A. Razeto, D. Reed, J. Refsgaard, T. Reilly, A. Renshaw, F. Retriere, E. Riccobene, D. Rigamonti, A. Rizzi, J. Rode, J. Romualdez, L. Russel, D. Sablone, S. Sala, D. Salomoni, P. Salvo, A. Sandoval, E. Sansoucy, R. Santorelli, C. Savarese, E. Scapparone, T. Schaubel, S. Scorza, M. Settimo, B. Shaw, S. Shawyer, A. Sher, A. Shi, P. Skensved, A. Slutsky, B. Smith, N. J. T. Smith, A. Stenzler, C. Straubel, P. Stringari, M. Suchenek, B. Sur, S. Tacchino, L. Takeuchi, M. Tardocchi, R. Tartaglia, E. Thomas, D. Trask, J. Tseng, L. Tseng, L. VanPagee, V. Vedia, B. Velghe, S. Viel, A. Visioli, L. Viviani, D. Vonica, M. Wada, D. Walter, H. Wang, M. H. L. S. Wang, S. Westerdale, D. Wood, D. Yates, S. Yue, V. Zambrano

**Affiliations:** 1 Nuclear Instruments S.R.L., Como 22045, Italy; 2 Elemaster Group S.p.A., Lomagna (LC) 23871, Italy; 3 Department of Physics, University of Houston, Houston, Texas 77204, USA; 4 Istituto per la Scienza e Tecnologia dei Plasmi, ISTP-CNR, Milano 20125, Italy; 5 TRIUMF, Vancouver, British Columbia V6T 2A3, Canada; 6 INFN Sezione di Pisa, Pisa 56127, Italy; 7 APC, Université de Paris, CNRS, Astroparticule et Cosmologie, F-75013 Paris, France; 8 SNOLAB, Lively, Ontario P3Y 1N2, Canada; 9 Gran Sasso Science Institute, L'Aquila 67100, Italy; 10 Istituto di Sistemi e Tecnologie Industriali Intelligenti per il Manifatturiero Avanzato, CNR STIIMA, Milano 20133, Italy; 11 Dipartimento di Fisica, Università di Pisa, Pisa 56124, Italy; 12 LSC, Laboratorio Subterráneo de Canfranc, Canfranc-Estación 22880, Spain; 13 JMP Solutions, London, Ontario N6N 1E2, Canada; 14 Dipartimento di Ingegneria Meccanica e Industriale, Università degli Studi di Brescia, Brescia 25123, Italy; 15 Dipartimento di Chimica e Chimica Industriale, Università di Pisa, Pisa 56124, Italy; 16 Camozzi Group S.p.A., Brescia BS 25126, Italy; 17 Dipartimento di Ingegneria Gestionale, dell'Informazione e della Produzione, Università di Bergamo, Bergamo, 24129, Italy; 18 INFN Sezione di Cagliari, Cagliari 09042, Italy; 19 Canadian Nuclear Laboratories, Chalk River K0J 1J0, Canada; 20 Fermi National Accelerator Laboratory, Batavia, Illinois 60510, USA; 21 INFN-CNAF, Bologna 40127, Italy; 22 INFN Sezione di Napoli, Napoli 80126, Italy; 23 CIEMAT, Centro de Investigaciones Energéticas, Medioambientales y Tecnológicas, Madrid 28040, Spain; 24 Dipartimento di Fisica, Università di Milano-Bicocca, Milano 20126, Italy; 25 Northwestern University, 633 Clark St, Evanston, Illinois 60208, USA; 26 Dipartimento di Scienze Economiche ed Aziendali, Università degli Studi di Cagliari, Cagliari 09042, Italy; 27 National Center for Innovative Technologies in Public Health, ISS (Italy National Institute of Health), Roma 00161, Italy; 28 Laboratoire Leprince Ringuet, École Polytechnique, Palaiseau, Cedex 91128, France; 29 Dipartimento di Fisica, Università degli Studi di Cagliari, Cagliari 09042, Italy; 30 Department of Physics and Astronomy, University of Rochester, Rochester, New York 14627, USA; 31 VEXOS, Markham, Ontario L3R 9X6, Canada; 32 INFN Sezione di Roma, Roma 00185, Italy; 33 Dipartimento di Fisica, Università degli Studi Federico II di Napoli, Napoli 80126, Italy; 34 Azienda Ospedaliera San Gerardo, Monza 20900, Italy; 35 Dipartimento di Medicina e Chirurgia, Università di Milano-Bicocca, Milano 20126, Italy; 36 Exida Canada Ltd., Mississauga, Ontario L5G 2B6, Canada; 37 INFN Laboratori Nazionali del Gran Sasso, Assergi (AQ) 67100, Italy; 38 Physics Department, Princeton University, Princeton, New Jersey 08544, USA; 39 Policlinico San Pietro, Ponte San Pietro 24036, Italy; 40 Department of Physics, Carleton University, Ottawa, Ontario K1S 5B6, Canada; 41 Arthur B. McDonald Canadian Astroparticle Research Institute, Kingston, Ontario K7L 3N6, Canada; 42 Department of Physics and Astronomy, University of Nebraska-Lincoln, Lincoln, Nebraska 68508, USA; 43 Max-Planck-Institut für Physik (Werner-Heisenberg-Institut), 80805 München, Germany; 44 Department of Physics and Astronomy, University of British Columbia, Vancouver, British Columbia V6T 1Z4, Canada; 45 ARAID, Fundación Agencia Aragonesa para la Investigación y el Desarrollo, Gobierno de Aragón, Zaragoza 50018, Spain; 46 Departamento de Medicina, Universidad de Zaragoza, Zaragoza 50009, Spain; 47 Department of APT, Faculty of Medicine, University of British Columbia, Vancouver V5Z 1M9, Canada; 48 CAPA (Centro de Astropartículas y Física de Altas Energías), Universidad de Zaragoza, Zaragoza 50009, Spain; 49 Dipartimento di Meccanica, Politecnico di Milano, Milano 20156, Italy; 50 Department of Respiratory and Anaesthesia Technology, Vanier College, Montréal, Quebec H4L 3X9, Canada; 51 Department of Physics, Engineering Physics and Astronomy, Queen's University, Kingston, Ontario K7L 3N6, Canada; 52 Dipartimento di Pediatria, Sapienza Università di Roma, Roma 00185, Italy; 53 School of Civil and Mechanical Engineering, Curtin University, Perth (Washington), Australia; 54 INFN Genova, Genova 16146, Italy; 55 Dipartimento di Fisica, Università degli Studi di Genova, Genova 16146, Italy; 56 Sergio Installazioni snc, Treviglio (BG) 24047, Italy; 57 INFN Sezione di Milano, Milano 20133, Italy; 58 Department of Computer Science and Engineering, Chalmers—University of Gothenburg, Gothenburg SE-412 96, Sweden; 59 DISIM, Università de L'Aquila, L'Aquila 67100, Italy; 60 Amherst Center for Fundamental Interactions and Physics Department, University of Massachusetts, Amherst, Massachusetts 01003, USA; 61 Equilibar L.L.C., Fletcher, North Carolina 28732, USA; 62 Dipartimento di Informatica, Universitá degli Studi di Milano, Milano 20122, Italy; 63 LPNHE, CNRS/IN2P3, Sorbonne Université, Université Paris Diderot, Paris 75252, France; 64 Istituto di Fisiologia Clinica del CNR, IFC-CNR, Pisa 56124, Italy; 65 INFN Sezione di Bologna, Bologna 40126, Italy; 66 SUBATECH, IMT Atlantique, Université de Nantes, CNRS-IN2P3, Nantes 44300, France; 67 St. Michael's Hospital, Unity Health Toronto, Ontario M5B 1W8, Canada; 68 12th Man Technologies, Garden Grove, California 92841, USA; 69 MINES ParisTech, PSL University, CTP-Centre of Thermodynamics of Processes, 77300 Fontainebleau, France; 70 AstroCeNT, Nicolaus Copernicus Astronomical Center, Polish Academy of Sciences, Warsaw 00-614, Poland; 71 Department of Medicine, University of British Columbia, Vancouver, British Columbia V6T 1Z4, Canada; 72 Department of Physics, University of Oxford, The Denys Wilkinson Building, Keble Road, Oxford OX1 3RH, United Kingdom; 73 Physics and Astronomy Department, University of California, Los Angeles, California 90095, USA

## Abstract

This paper presents the Mechanical Ventilator Milano (MVM), a novel intensive therapy mechanical ventilator designed for rapid, large-scale, low-cost production for the COVID-19 pandemic. Free of moving mechanical parts and requiring only a source of compressed oxygen and medical air to operate, the MVM is designed to support the long-term invasive ventilation often required for COVID-19 patients and operates in pressure-regulated ventilation modes, which minimize the risk of furthering lung trauma. The MVM was extensively tested against ISO standards in the laboratory using a breathing simulator, with good agreement between input and measured breathing parameters and performing correctly in response to fault conditions and stability tests. The MVM has obtained Emergency Use Authorization by U.S. Food and Drug Administration (FDA) for use in healthcare settings during the COVID-19 pandemic and Health Canada Medical Device Authorization for Importation or Sale, under Interim Order for Use in Relation to COVID-19. Following these certifications, mass production is ongoing and distribution is under way in several countries. The MVM was designed, tested, prepared for certification, and mass produced in the space of a few months by a unique collaboration of respiratory healthcare professionals and experimental physicists, working with industrial partners, and is an excellent ventilator candidate for this pandemic anywhere in the world.

## I. INTRODUCTION

Mechanical ventilation is a necessary tool in every modern intensive care unit (ICU). The type and intensity of ventilation support required by a patient vary over the course of treatment. Modern mechanical ventilators are versatile and adapt to patient needs. Commercially available devices control the volume, pressure, or gas flow and the breathing cycle timing. They support patients who cannot breathe and who can still trigger a mechanical cycle by a spontaneous inspiratory effort.^1^ Present-day mechanical ventilators are complex machines, consisting of many specialized components and featuring several ventilation modes.^1–3^

The exponential growth of COVID-19 in 2020 put ICUs all over the world under unprecedented pressure. The drastic increase in demand of these devices exceeded the capacity of the existing supply chains, especially in regions where cross-border supply has been disrupted. This created the need for a simpler, but technically suitable machine that could be mass produced on a very large scale and in a short timeframe.

The MVM collaboration has responded to this need by developing the Mechanical Ventilator Milano (MVM), a reliable, fail-safe, and easy to operate mechanical ventilator, built from a small number of readily available parts.

The design is inspired by the idea proposed by Manley^4^ back in 1961, i.e., *the possibility of using the pressure of the gases from the anesthetic machine as the motive power for a simple apparatus to ventilate the lungs of the patients in the operating theater,*^5^ but using a completely different design, i.e., in particular, replacing all moving mechanical parts with electro-mechanical components, allowing better parameter control, and improving robustness and reliability in the long-term operation, often needed by COVID-19 patients, as also discussed in Sec. II.

The MVM was designed in collaboration between healthcare professionals and experimental physicists, benefiting from the medical expertise of the former and the latter's technical expertise in designing gas handling systems, with industrial partners (Elemaster, Italy, and Vexos, Canada) who provided access to laboratories and production lines for both R&D and prototype construction.

The MVM was certified by the Center for Devices and Radiological Health, U.S. Food and Drug Administration (FDA) for Emergency Use Authorization in May 2020, *in response to concerns related to insufficient supply and availability of FDA-cleared ventilators for use in healthcare settings to treat patients during the COVID-19 pandemic*, and received Health Canada Medical Device Directorate Authorization *for Importation or Sale, under Interim Order for Use in Relation to COVID-19* in September 2020. A production run of 6000 units was recently performed in Canada (Vexos and JMP Solutions). The cost of a single unit turned out to be about 10000 US$, about five times less than commercially available mechanical ventilators for ICUs.

The MVM is a mechanical ventilator for adult patients assisted with tracheal tubes, designed to control pressure, while the resulting delivered volume is measured. Pressure control is widely used for COVID-19 patients, who are susceptible to further lung damage from too high pressure or volume.^6–8^ The MVM can be operated in two modes, pressure-controlled ventilation (PCV) and pressure-support ventilation (PSV). In PCV mode, the ventilator controls the timing of the breathing cycle and regulates the pressure applied to the patient. PCV mode is used in the acute phase of the disease when patients are deeply sedated or paralyzed. By delivering the mechanical breath with an exponentially decelerating flow pattern, PCV allows pressures to balance across the lung units during a preset time, resulting in significantly reduced pressures and in improved distribution of ventilation. This lowers the risk of barotrauma attributable to the high pressures often required to ventilate these patients.^9^ PSV is an assisted ventilatory mode that is patient-triggered, pressure-limited, and flow-cycled. The main use of this mode is for the weaning of the patient from mechanical ventilation because it unloads the work of breathing and allows a gradual decrease in ventilator support until extubation.^10^

Invasive mechanical ventilation exposes the patient to risks arising from infections, pneumothorax, ventilator-associated lung injury, and oxygen toxicity,^11^ as well as from operator error. Therefore, the MVM has a sophisticated integrated alarm system, in accordance with EN 60601–1-8:2007, which monitors the various aspects of the breathing cycle and alerts the operator when any anomaly arises. The hardware and software are designed to be as straightforward as possible to mitigate the risk of operator error. In addition, the MVM must be used in association with an oximeter and a capnometer.

Vocabulary and semantics are defined consistent with ISO (International Organization for Standardization) 19223:2019. The system is designed to comply with the guidelines defined in ISO 80601–2-12:2020. The test results demonstrating compliance are discussed in Sec. VI A.

## II. MEDICAL CONSIDERATIONS

According to current studies, approximately 5% of patients hospitalized with COVID-19 develop severe lung damage.^12,13^ This condition reflects the pathophysiology of severe acute respiratory distress syndrome (ARDS). ARDS is a disease characterized by reduced lung compliance due to the loss of surfactant function, collapsed lung areas, and accumulation of interstitial/alveolar plasma leakage. Computed Tomographic (CT) scans demonstrate uneven distributions of aerated areas and dense, consolidated regions of the lungs; the remaining alveolar surface for gas exchange is greatly reduced in adult patients, a condition termed baby lung.^14^ It has been suggested that the clinical management of COVID-19 patients with severe lung damage should follow the established guidelines for ARDS subjects.^15,16^ This opinion has been confirmed by a recent study comparing COVID-19 subjects with patients affected by ARDS due to other causes; the physiological differences between ARDS from COVID-19 and other causes were found to be small.^12^ The principal supportive treatment for ARDS patients is mechanical ventilation with supplemental oxygen, currently deemed most appropriate, following a discussion that has been ongoing since the syndrome was first described in 1967.^17^ The tidal volume ($V_{\mathrm{tidal}}$) is a key parameter, with potentially unfavorable effects if incorrectly set, such as ventilator-induced lung injury. Starting in the 1970s, a $V_{\mathrm{tidal}}$ value of 12–15 ml per kg of predicted body weight (PBW) was recommended by clinicians until, in 2000, the Acute Respiratory Distress Syndrome Network reported that the length of hospital stay and mortality could be significantly reduced using a lung-protection strategy. This strategy includes a low $V_{\mathrm{tidal}}$ ventilation (8 ml per kg of PBW) to avoid overdistension of the baby lung, a limited plateau pressure (PP) of ≤30 cm H_2_O, and a sufficient positive end expiratory pressure (PEEP) of ≤15 cm H_2_O.^18^ PEEP targeting must be tailored to prevent lung injury due to cyclic alveolar opening and collapse and to improve oxygen delivery (amount of lung recruited) while avoiding volutrauma (lung overdistension) and cardiovascular compromise.

In addition to invasive ventilation, a series of therapies were tested in patients affected by COVID-19 pneumonia: nasal high-flow therapy, continuous positive airway pressure, and noninvasive ventilation. It has been found that these strategies are suitable only in the mild, early stage of the disease, when they may be effective in stabilizing the clinical course. The early stage of COVID-19 pneumonia is mainly characterized by an injury to the vascular endothelium, disrupted vasoregulation, and hypoxemia owing to ventilation-perfusion mismatch. The greater part of the lung is not yet affected, which explains the relatively good pulmonary compliance at this stage and the interstitial rather than alveolar edema seen in the CT scans.^16^ For patients with severe cases or who do not respond well to milder early stage treatments, COVID-19 pneumonia may develop into ARDS, requiring treatment with an invasive ventilation device, such as the MVM. The length of invasive ventilation treatment can last from a few days to several weeks and depends on the severity of ARDS and the presence or absence of comorbidities.^19^

## III. VENTILATOR DESIGN

**FIG. 1.**
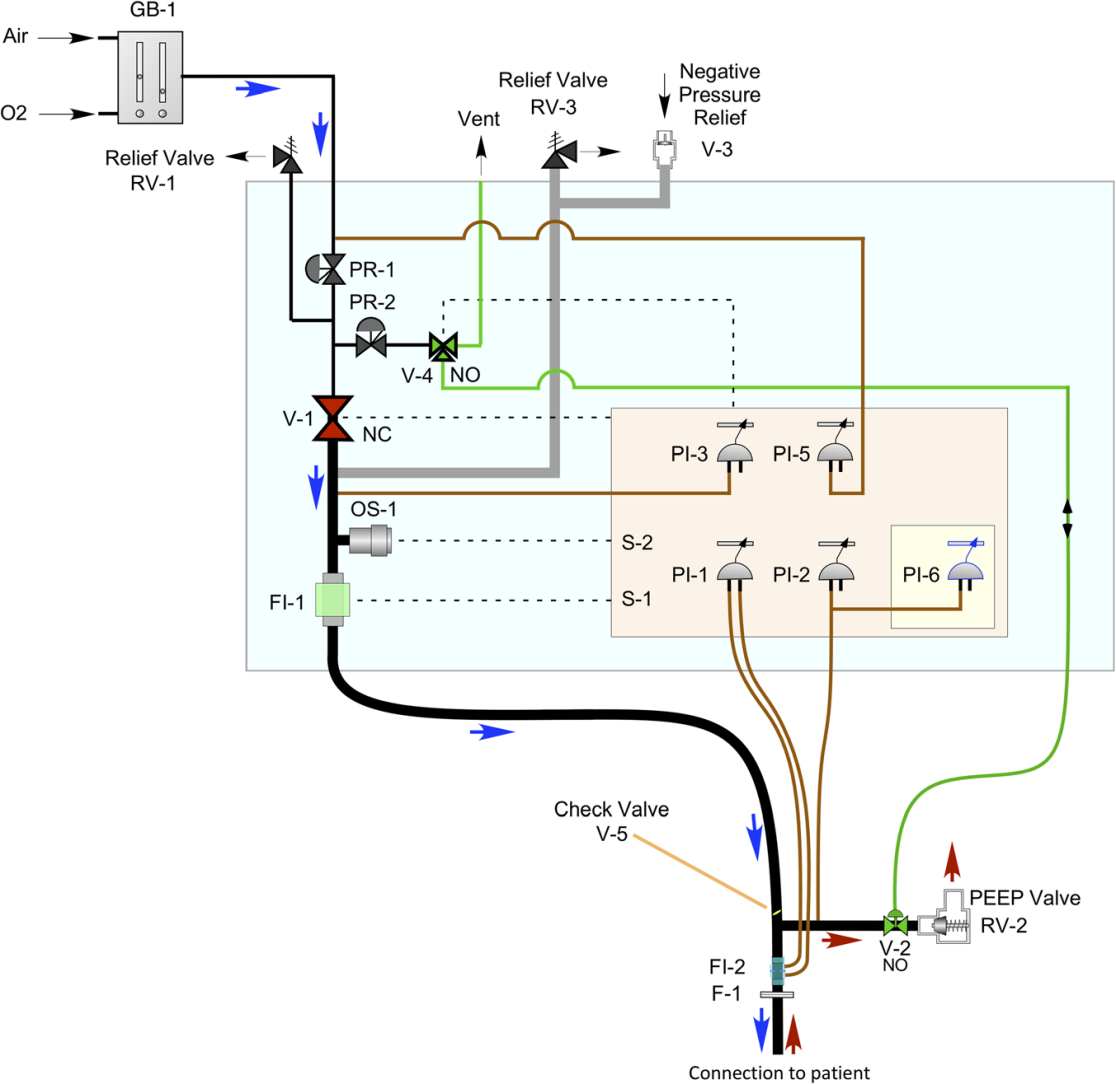
A schematic of the MVM ventilator system (light blue box) with the connection to the patient. Dashed lines indicate electrical connections, and solid lines indicate gas connections. Thick black lines represent the breathing circuit, thin red lines are connections to pressure measurements, and the green line is the gas connection to drive the pneumatic valve at the end of the expiratory line. The direction of gas flow is indicated by the blue (inspiratory phase) and red (expiratory phase) arrows. The lines in gray indicate the breathing circuit relief lines. The beige rectangle represents the main electronics and control board, and the yellow square represents the supervisor board, which provides a redundant monitor and control.

Figure 1 shows a schematic of the MVM, with typical connections to the patient and to the oxygen and medical air lines. The gas blender, GB-1, is external to the MVM unit. The breathing circuit and other items that get in contact with or are near the patient are replaced before each use. The ventilator receives its breathing supply from the facility, and the operator sets the fraction of inspired oxygen (FiO_2_), i.e., the concentration of oxygen in the gas mixture, on the external gas blender GB-1. The pressure out of the gas blender is monitored by PI-5 and regulated to the pressure appropriate for the MVM by PR-1. RV-1 relieves in the case of excess pressure at the input to the MVM.

The MVM operates by opening the inspiratory valve, V-1, to provide the breathing gas to the patient at the desired pressure with the expiratory valve, V-2, closed. On command from the controller, V-1 is then closed and V-2 is opened, to allow the patient to exhale. A mechanical PEEP valve, RV-2, sets a positive end-expiratory pressure. At the end of the expiratory phase, V-2 is closed, V-1 is opened, and the cycle repeats.

V-1 is a proportional solenoid valve controlled by a loop using the pressure measured by PI-3. V-2 is a low-impedance pneumatically operated valve and is controlled by a three-way electrical valve, V-4. To avoid the need for a second source of gas, the pneumatic control is reached using the MVM input gas regulated to low pressure by PR-2. Relief valves RV-3 and V-3 prevent over-pressure and under-pressure, respectively, in the line to the patient. The pressure in the expiratory line is measured by two independent indicators PI-2 on the main control board and PI-6 on the supervisor board. The (unidirectional) flow of gas to the patient from V-1 is measured by FI-1; the (bidirectional) flow of gas into and out of the patient is indicated by PI-1, using the pressure-differential developed over FI-2. The oxygen content of the gas provided to the patient is measured with OS-1. The breathing circuit shown is a standard single-limb unit with integrated wye. V-5 is a check valve to prevent backflow from the patient into the MVM.

F-1 is a bacterial filter ensuring that the air exhausted from the ventilator is free from bacteria or virus particles and, thus, safe for doctors and nurses surrounding the patient. Indeed, this filter prevents the large diffusion of droplets that would, otherwise, be emitted in the surroundings, as demonstrated by many flow visualization studies.^20–22^ It should be added, however, that none of these studies has targeted the surroundings of an infected mechanically ventilated patient, which is a potential subject for a new study.

**FIG. 2.**
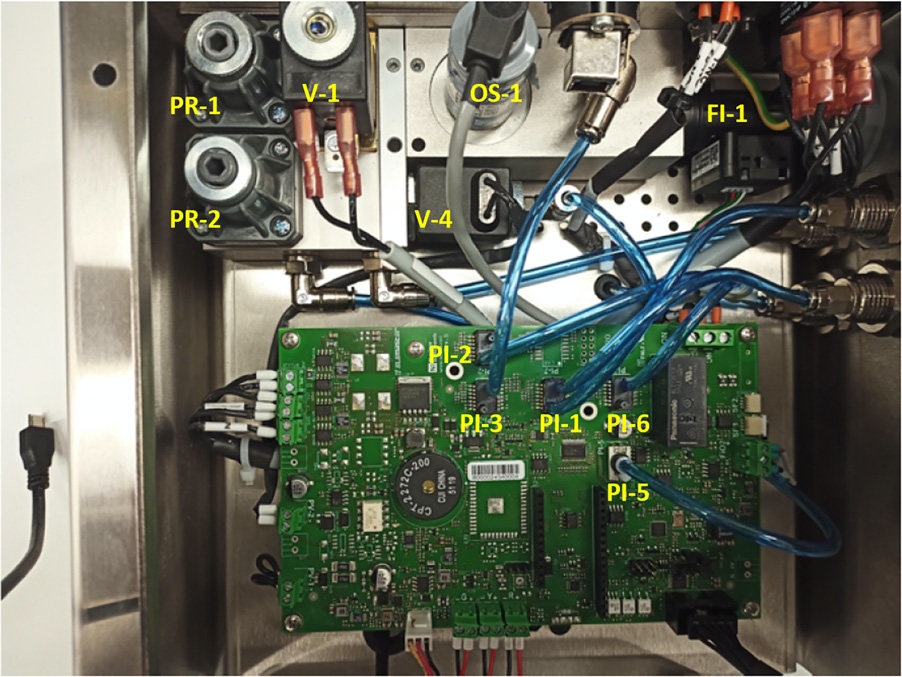
A view of the inside of the MVM: the labels identify the components shown in Fig. 1.

Figure 2 shows the inside of the stainless steel enclosure of the MVM, with the pneumatic control components and the electronics boards.

## IV. OPERATING MODES

### A. Pressure-controlled ventilation mode

PCV is a time-cycled ventilation mode in which the operator sets the inspiratory pressure, the PEEP, the duration of the inspiratory phase of the breathing cycle, and the number of breaths per minute. As the flow and volume are not directly set, the resultant patient tidal volume varies depending on lung compliance and resistance, patient effort, and inspiratory pressure.

A new inspiration begins either after a breathing cycle is completed according to the set respiratory rate (RR) or if the MVM detects initiation of a breath by the patient before the cycle completes and the inhale trigger criteria are met. The trigger window for a patient-initiated breath occurs during the expiratory phase of the previous breath. When inspiration begins, the MVM provides the patient with the set inspiratory pressure ($P_{\mathrm{insp}}$) for the set duration of the inspiratory phase of the breathing cycle. The respiratory rate and the ratio between the inspiratory and expiratory times (I:E) are the parameters that control the time cycle.

### B. Pressure-support ventilation mode

In PSV mode, the MVM provides pressure to help the patient breathe, while the patient controls the RR. This mode is unsuitable for patients who cannot initiate breaths on their own. A pressure-support breath is initiated when the MVM detects a sudden pressure drop, which indicates the start of patient inspiration. Such sudden drops in the pressure are determined by measuring the changes in the rate at which the pressure is decreasing, indicated by the downward curvature near the start of the pressure vs time waveform.

When a pressure-support breath is triggered, the MVM increases the pressure to the set $P_{\mathrm{insp}}$. When the patient's inspiratory flow drops below 30% of its peak, the MVM ends inspiration and returns the pressure to the baseline, allowing exhalation. If the patient does not trigger a breath within a set apnea-trigger time window, the MVM switches to PCV mode, and an apnea alarm is activated, that the operator must reset.

## V. ELECTRONICS AND SOFTWARE

The electronics and software are responsible for controlling the valve systems, reading the pressure, flow, and oxygen sensors, generating audible and visible alarms in the hardware (including LEDs, and buzzers), monitoring the correct ventilation, and interacting with the operator.

Both electronics and software are composed of three main macro-components: graphical user interface (GUI), controller, and supervisor. The GUI is a touch screen panel that displays the information needed to check the respiratory condition, allows parameter setting, and displays ventilation parameters and alarm settings. When the controller receives operator input from the GUI, it communicates with the valve controllers, serial interfaces, and other subcomponents and sends them commands. The supervisor monitors the overall system behavior and ensures that the machine operates safely.

### A. Electronic hardware

The MVM operations are managed by an electronic board hosting all the components required to measure the relevant quantities, drive the solenoid and proportional valves, and activate visual and audio signals for the operator. The board houses a micro-controller (Espressif-ESP32), a Raspberry Pi 4, and the supervisor.

The ESP32 includes a dual-core 240 MHz micro-controller, 0.5 MB of RAM, Wi-Fi, and Bluetooth connectivity. The ESP32-based solution is widely used in the Internet of Things environment, and hence, it is readily available. It is programed using an Arduino core (Espressif). A USB connection between the main controller and the Raspberry Pi enables the transmission of commands and settings from the GUI to the ESP32 and the read-back of the system status.

The supervisor implements a micro-controller that is programed with the standard Arduino boot loader to allow firmware updates via an opto-isolated serial connection with the Raspberry Pi. This connection is also used to enable monitoring during the ventilation.

The power is provided by an external unit, equipped with a battery ensuring 2 h of autonomy, which generates two independent 12 V sources. One is regulated with step-down converters to 3.3 V and 5 V, as required to operate the sensors and the ESP32. The other one provides power to the valves and to the supervisor and Raspberry Pi. A failure of either of the supply lines would still leave a micro-controller active to alert the operator and to return to a safe state.

Three I2C buses connect the sensors and the micro-controllers. The main I2C bus connects the pressure sensors PI-1 and PI-2 and the flow meter FI-1 to the ESP32. Two ADCs connect to the main bus, digitizing the readings from the FiO_2_ analog oxygen sensor and the PI-5 analog pressure sensor and monitoring internal voltages. The main I2C bus connects the supervisor to the main processor, enabling the watchdog function. An I2C bus multiplexer is installed to avoid address conflicts. A priority bus connects the PI-3 sensor to the ESP32 and allows it to be polled at frequencies over 1 kHz, as required by the fast proportional–integral–derivative pressure controller. A third dedicated I2C bus connects the supervisor with the PI-6 sensor and an ADC that monitors the board's internal voltages. This auxiliary bus ensures normal supervisor operation in case the main I2C bus freezes.

The control boards include ON/OFF valve controls, current-feedback valve controllers, and visual and audio alarm circuits.

### B. Software

**FIG. 3.**
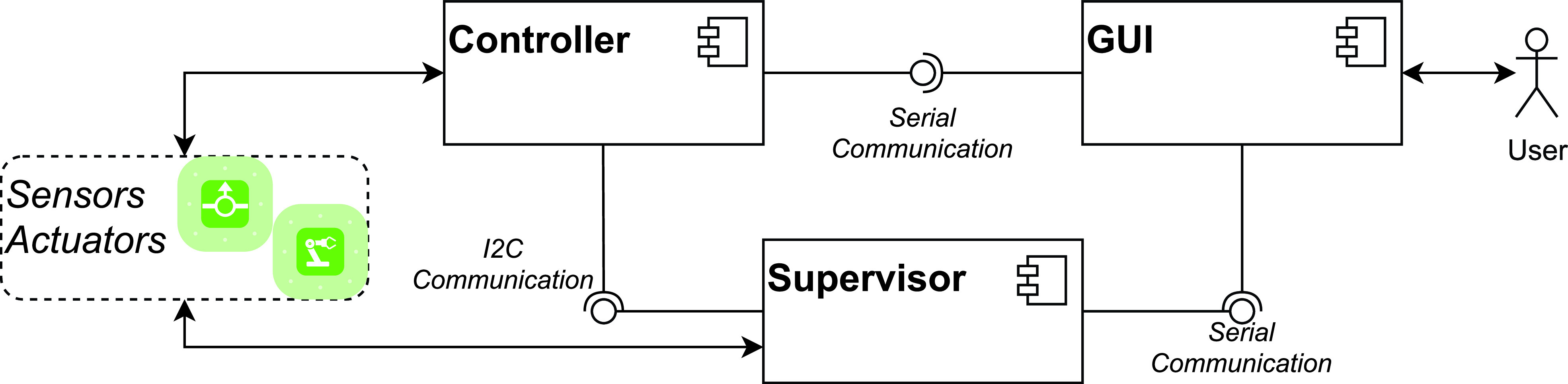
The high-level software architecture.

The high-level software architecture, shown in Fig. 3, illustrates the communication among the three software components.

#### 1. GUI

**FIG. 4.**
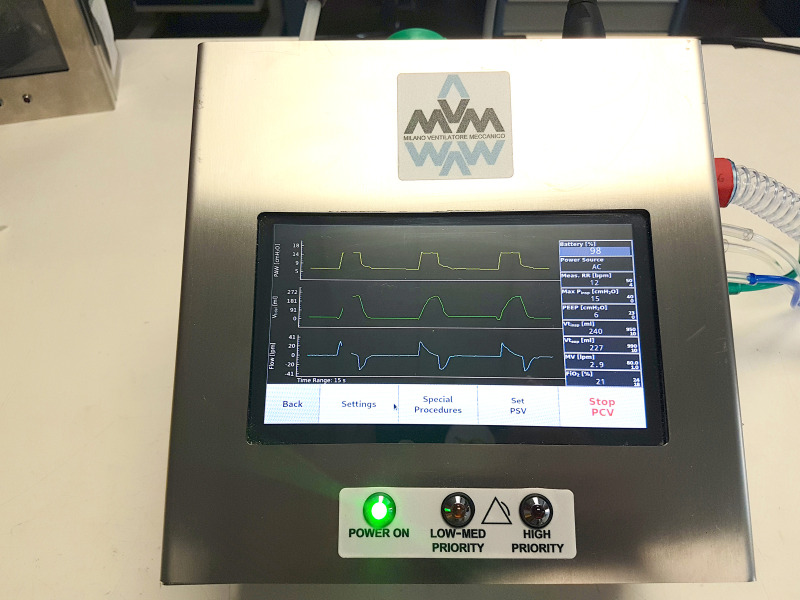
Front view of the MVM.

When turned on, the GUI guides the operator through startup procedures, including setting operating parameters and alarm thresholds, and performing hardware and software tests. The GUI home screen has three parts, as shown in Fig. 4. The center part displays the three monitored parameter waveforms (airway pressure, inspiratory tidal volume, and airflow). A side panel displays other monitored parameters, alarms, and warnings, while the bottom part is dedicated to parameter setting. The GUI is written in Python3 using the PyQt5 library. It runs on a Raspberry Pi, chosen for its wide availability and its computing power to power consumption ratio.

#### 2. Controller

The controller software, implemented in C++, receives input from the GUI and interacts directly with the hardware by receiving patient breathing data and issuing commands. The controller software is divided into four components: the interface, monitor, control, and hardware driver. The interface communicates with the GUI and supervisor, the monitor observes the sensors and the system status and triggers alarms, the control changes the respiratory phases using a state machine and controls valves, and the hardware driver opens and closes valves and raises the visual and audio alarms.

**FIG. 5.**
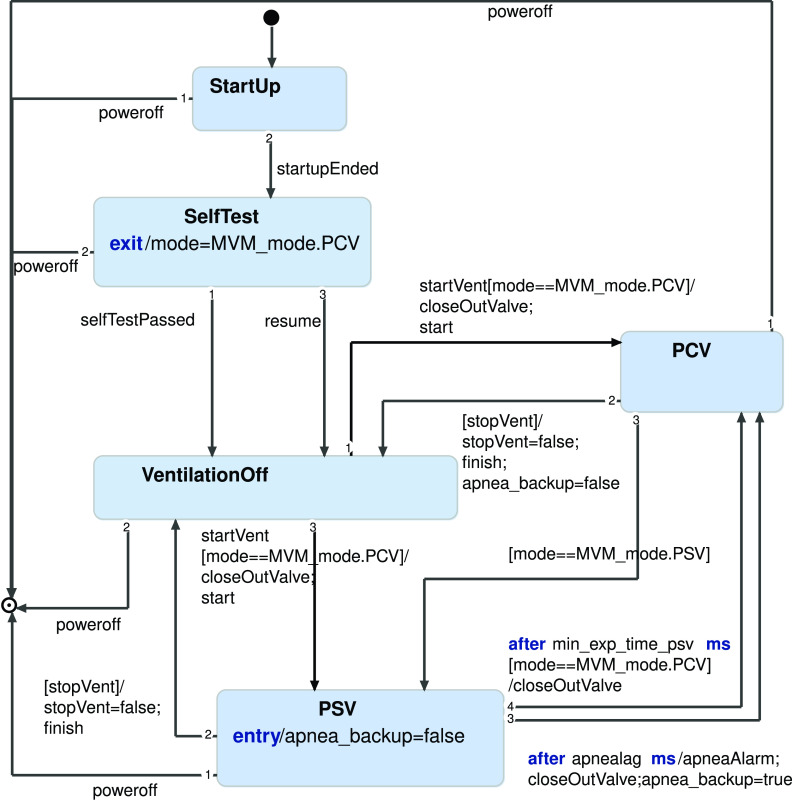
The state machine of the controller software.

The operation modes are implemented in the controller with a state machine. Figure 5 shows a simplified version of it, modeled using the Yakindu Statechart Tool (Itemis AG). It describes the states in which the device can operate and the transitions between them. In particular, the machine starts in the StartUp state and needs to complete all the Self-Tests before operating. The state machine interacts with the valve controller, opens and closes the expiratory valve V-2, and sets the desired pressure for inspiratory valve V-1. The corresponding C++ code has been generated from the statechart model and integrated into the controller logic. The Self-Tests take about ten minutes to complete, assuming that the doctor already knows the parameters to be set for the alarm thresholds and has been using the MVM before.

In a breathing cycle, the MVM controls valve V-1 to increase the pressure during the inspiration phase for the prescribed time duration. The actual tidal volume depends on the patient response and on how quickly the regulator reaches the pressure set point, $P_{\mathrm{set}}$. To protect the patient, the device must never overshoot this point significantly, by controlling the pressure peaks. To satisfy these objectives and constraints, the control algorithm has been designed to reach $P_{\mathrm{set}}$ in a fixed rise time and without overshooting.

**FIG. 6.**
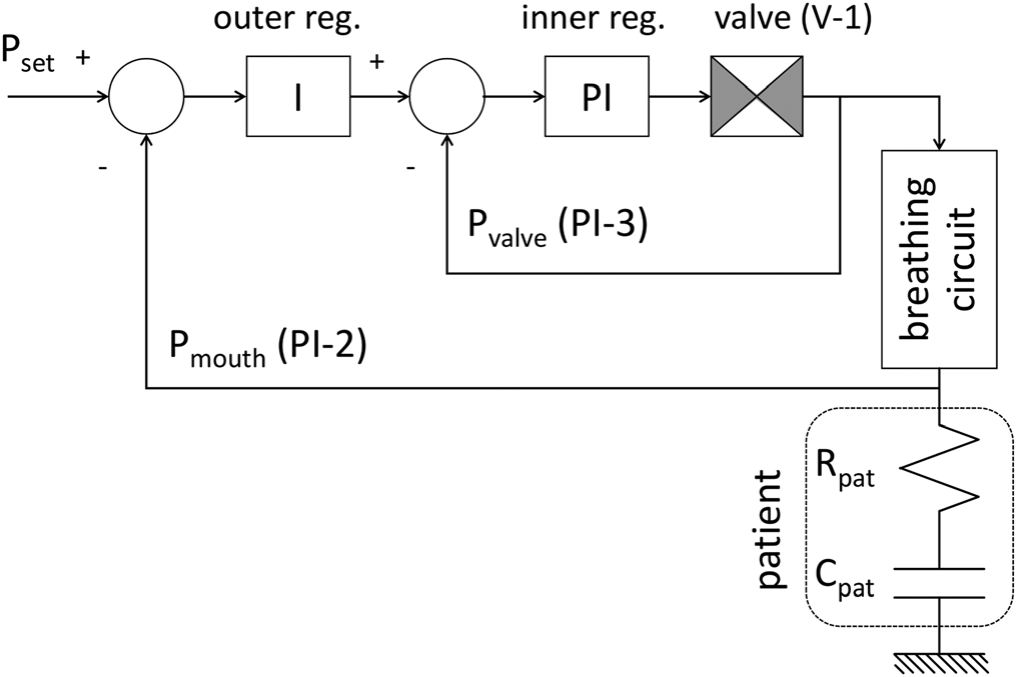
Schematics of the pressure controller that manages the pressure at the patient. It is based on two nested loops. The Proportional-Integral regulator of the inner loop, driven by the difference between the output of the outer loop regulator and the pressure at valve V-1 output (PI-3 sensor), actuates the V-1 valve. The Integral regulator of the outer loop ensures that the pressure at the patient (sensor PI-2) tracks the set pressure value. Pressure sensors PI-3 and PI-2 are placed at the input and output of the breathing circuit connecting the ventilator to the patient, respectively. The controller automatically identifies a simplified model of patient lungs to tune the inner loop regulator.

A control architecture that implements two nested loops, often used in safety-critical industrial products, allows a simpler controller tuning, based on first-principle considerations, and ensures a sufficient robustness against disturbances. Figure 6 shows the controller structure and the simplified lumped parameter model used to represent the patient respiratory system, i.e., the lung linear resistance, $R_{\mathrm{pat}}$, and compliance, $C_{\mathrm{pat}}$. The outer loop regulates the pressure at the patient with the PI-2 sensor, read at a rate of 300 Hz; its controller is a simple integrator, whose time constant depends only on the desired rise time. The inner loop controller is a proportional-integral regulator that is automatically tuned via the patient parameters. The inner loop is fed back with the pressure at valve outlet PI-3, read at a rate of 1 kHz. The patient parameters are automatically determined during the first three respiratory cycles. To ensure patient safety during this phase, the inner controller parameters are fixed and set to guarantee smooth pressure rising within 300–500 ms, over the expected range of $R_{\mathrm{pat}}$ and $C_{\mathrm{pat}}$, with an overshoot always lower than 2 mbar. A recursive least-squares method is used to estimate the patient parameters, limiting memory use in the micro-controller. The estimator uses pressure and flow measurements from a single inhalation, assuming the lumped-parameter model and negligible ventilator resistance and compliance compared to the lungs. The identified $R_{\mathrm{pat}}$ and $C_{\mathrm{pat}}$ are used to tune the inner loop and the time constant of the outer loop to reach a more desirable rise time of about 100 ms. Model parameters, estimated after three respiratory cycles, are continuously updated and re-tuned as necessary.

#### 3. Supervisor

The supervisor software is implemented in C++. It is responsible for monitoring the controller, the GUI, and the hardware. In the case of errors, it raises alarms if not already raised by the controller or the GUI, ensuring patient safety. For instance, if the pressure in the circuit exceeds the maximum allowed value for a given duration, the supervisor switches off the ventilation and brings the valves into the safe position (valve V-1 closed and V-2 open). Like the controller, the supervisor has a state machine that models its behavior. After startup, the supervisor waits for the operator to start the self-test procedure. Then, the supervisor alternates between two operation modes: breathing off (the MVM is ready to work, but it is not ventilating) and breathing on (the device is ventilating). The supervisor can move into a fail-safe state from any state, in the case of errors.

### C. Software certification

**FIG. 7.**
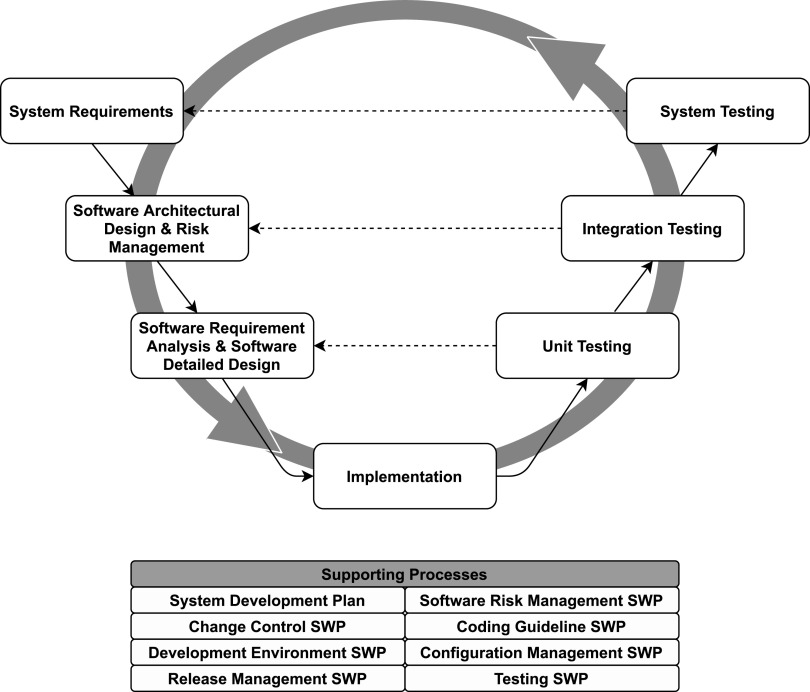
Software development process. It is based on the V-model, and agile practices have been integrated (circular arrow) to facilitate team collaboration, iterative development, and flexible response to changes. Each development activity on the left-hand side of the V-model corresponds to a testing activity on the right-hand side.

Software for mechanical ventilators must comply with the IEC 62304:2015, a global benchmark for the management of the software development lifecycle. The standard was prepared jointly by the IEC (International Electrotechnical Commission) and the ISO technical committees. It is recognized by many organizations, including the European Union, the U.S. FDA, ANSI (American National Standard Institute), and the SFDA (State Food and Drug Administration) of China and Japan. To comply, we have applied the *V-model*, which involves the development process shown in Fig. 7. The GUI and controller software were required to be in safety class A and the supervisor software in safety class C.

## VI. TESTING

### A. Tests in PCV mode, based on the ISO testing protocol

The test setup is equivalent to that described in Fig. 201.102 in the ISO 80601–2–12:2020 reference standard. The breathing simulator (IngMar Medical-ASL 5000) is used both as a test lung (with settable compliance and resistance) and as an independent sensor for pressure, flux, temperature, and oxygen concentration.

This series of tests refers to the ISO reference standard, Section 201.12, for pressure-controlled inflation-type testing, Subsection 201.12.1.102, verifying that breathing parameter values set and measured by the MVM agree to within the declared accuracy range. For the tidal volume, $V_{\mathrm{tidal}}$, which is not set in MVM, the measured value is compared with the value independently measured by the breathing simulator.

**FIG. 8.**
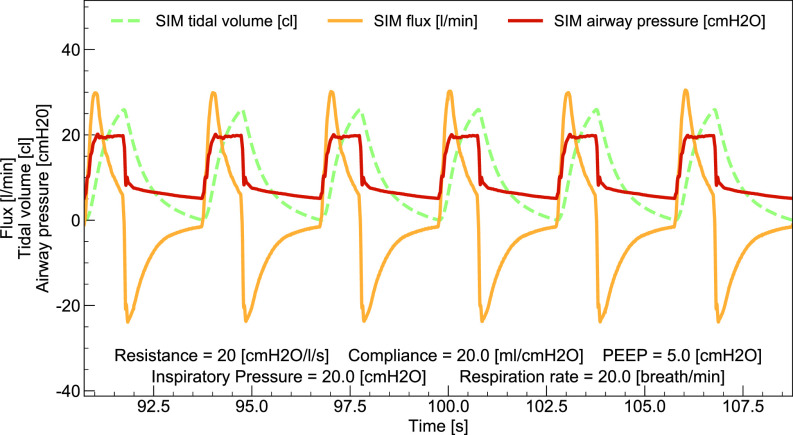
Example waveforms from the breathing simulator with the MVM in PCV mode, referring to test number 5 of Table 201.105 of the ISO 80601-2-12:2020 standard.

Table I summarizes acceptable ranges for the measured breathing parameters of interest. Out of the 21 tests in ISO standard Table 201.105, we performed the first 11, as they are the ones involving tidal volumes in the range relevant for MVM operation ($50\;\text{ml}\leq V_{\mathrm{tidal}}\leq 500\;\text{ml}$). For each condition, we ensured that the breaths resulted in smooth and reproducible time traces, as shown in Fig. 8. Measurements were taken over 30 cycles in steady-state conditions. The airway PP and PEEP were measured in the last 50 ms of the inspiratory and expiratory phases, respectively. $V_{\mathrm{tidal}}$ was obtained by averaging the MVM measurement over 30 cycles and compared with breathing simulator measurements, averaged over the same cycles. RR and I:E were calculated from the collected waveforms using custom algorithms. The calculated values agree well with those from the breathing simulator. When comparing the breathing simulator measurements with the values set on MVM (or in the case of $V_{\mathrm{tidal}}$ with the MVM reported value), all breathing parameter values are found to be within the acceptable range of Table I, individually for all the 30 cycles considered in each of the 11 tests.

**TABLE I.** Acceptable ranges for the measured breathing parameters of interest. BAP stands for the Baseline Airway Pressure.

Parameter	Range	Units
PP	±[2+(4 % of set value)]	(cm H_2_O)
PEEP	±[2+(4 % of set BAP value)]	(cm H_2_O)
$V_{\mathrm{tidal}}$	±[4+(15 % of measured value)]	(ml)
RR	±[0.5+(5 % of set value)]	(${\text{min}}^{-1}$)
I:E	±[0.1+(5 % of set value)]	

### B. Single-fault test condition based on ISO protocols

The system robustness against the specific single fault conditions listed in Section 201.13.2.101 of the ISO standard was successfully tested. The test conditions relate to the disruption, disconnection, or bad connection of the external components, such as the gas delivery to the patient pathway or the pressure to the patient sensors. This test ensures that when any one of the fault conditions is triggered, the system successfully keeps the patient breathing parameters in the safety zone and triggers an alarm. Fault conditions are induced manually by disconnecting external components during test operation, such as the PI-2 sensor. Once the default conditions are restored, the ventilator performance is reestablished automatically.

### C. Long-term durability tests

Several units were tested continuously for three month periods. During this testing, the units met all the criteria defined for correct operation as there were no alarms of any kind recorded during the testing.

### D. Response of the ventilator to an increased oxygen concentration

Paragraph 201.12.1.105 of the ISO standard requires evaluating the ventilator's speed of response to a change in the FiO_2_ set point. This test involves measuring $t_{\text{rise}}$, the time required for the oxygen concentration in the lungs to rise from 21% to 90% when the input FiO_2_ is suddenly increased to 100%. This test is performed by connecting the MVM to a gas analyzer (FLUKE-VT900), which measures FiO_2_, and to an adjustable test lung, loading the parameters in Table II into the MVM, and starting it in PCV mode with FiO_2_ at 21%. Once the steady-state conditions are reached, FiO_2_ is then abruptly increased to 100%. The measured time $t_{\text{rise}}$ for the oxygen concentration to reach 90% during the expiratory phase is reported in Table II.

**TABLE II.** Parameters used for testing the MVM response to the increase in FiO_2_ and the resulting $t_{\text{rise}}$ measurements.

$V_{\mathrm{tidal}}$	I:E	RR	$R_{\mathrm{pat}}$	$C_{\mathrm{pat}}$	$t_{\text{rise}}$
(ml)		(${\text{min}}^{-1}$)	[cm H_2_O/(Ls)]	[ml/cm H_2_O)]	(s)
500	1:2	10	5	20	76
150	1:2	20	20	10	85

### E. Tests in pressure-supported ventilation mode

In PSV mode, the patient actively initiates a breathing cycle by producing a pressure decrease in the airway. The ventilator must readily recognize this decrease and provide airflow support so as not to stress the patient's respiratory system. The MVM must also recognize the patient-driven end of the inspiratory phase and begin the expiratory phase. To achieve a quick response to the recognition of the patient breathing effort, a trigger system based on the second derivative of the airway pressure with respect to time was devised. The trigger sensitivity is set by varying the threshold on the maximum value of this parameter.

**FIG. 9.**
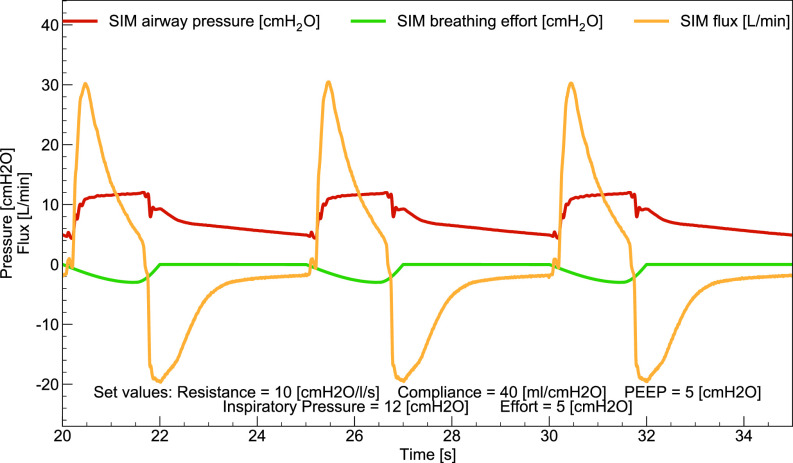
Example waveforms from the breathing simulator with the MVM in PSV mode.

A typical waveform of the MVM operating in PSV mode is shown in Fig. 9. Even in the case of a patient's breathing effort as low as 2 cm H_2_O, the MVM recognizes the effort within 100–200 ms and starts the inspiratory phase providing the desired pressure support. This is well within the parameters required by physicians for respiratory rehabilitation.

### F. Evaluation of biocompatibility

Biocompatibility of breathing gas pathways of a brand-new MVM was assessed according to the ISO 18562–3:2017 guideline. The analyses were carried out using a thermal desorption unit coupled to gas chromatography and mass spectrometry detection, in compliance with the ISO 16000–6:2011 guideline. The tests showed that volatile emissions from a brand-new MVM system are limited to a few chemicals, which mainly belong to the siloxanes family. The presence of such chemicals was somehow expected, due to the large use of silicones in biomedical devices. All values complied with permissible levels suggested from ISO 18562–3:2017 and decreased after one day of use as they were washed away from medical air.

## VII. CONCLUSION

The Mechanical Ventilator Milano, a novel intensive therapy mechanical ventilator designed for rapid, large-scale, low-cost production for the COVID-19 pandemic, was conceived, designed, prototyped, and tested by a unique international collaboration of scientists, medical specialists, and industrial partners. Within few months, it has received certification from U.S. and Canadian health agencies for interim use during the COVID-19 pandemic, representing an achievement that we hope will save lives. The information contained in this and our previous papers is open for access and available for use under the terms of CERN Open Hardware License v2.0 type “P.” For more detailed engineering and manufacturing information, please contact the manufacturers.

## Data Availability

The data that support the findings of this study are available from the corresponding author upon reasonable request.
